# Association of 25-hydroxyvitamin D with risk of overall and colorectal cancer among Japanese using a Mendelian randomization approach

**DOI:** 10.1038/s41598-023-29596-8

**Published:** 2023-02-10

**Authors:** Ryoko Katagiri, Atsushi Goto, Shiori Nakano, Masahiro Nakatochi, Yuriko N. Koyanagi, Masao Iwagami, Akiko Hanyuda, Taiki Yamaji, Norie Sawada, Yohko Nakamura, Sho Nakamura, Kiyonori Kuriki, Sadao Suzuki, Issei Imoto, Yukihide Momozawa, Isao Oze, Hidemi Ito, Shoichiro Tsugane, Kenji Wakai, Keitaro Matsuo, Motoki Iwasaki

**Affiliations:** 1 Division of Epidemiology, National Cancer Center Institute for Cancer Control, Tokyo, Japan; 2 Department of Health Data Science, Graduate School of Data Science, Yokohama City University, Yokohama, Kanagawa Japan; 3 Public Health Informatics Unit, Department of Integrated Health Sciences, Nagoya University Graduate School of Medicine, Nagoya, Aichi Japan; 4 Division of Cancer Information and Control, Aichi Cancer Center, Nagoya, Aichi Japan; 5 Department of Health Services Research, University of Tsukuba, Tsukuba, Ibaraki Japan; 6 Department of Ophthalmology, Keio University School of Medicine, Tokyo, Japan; 7 Division of Cohort Research, National Cancer Center Institute for Cancer Control, Tokyo, Japan; 8 Cancer Prevention Center, Chiba Cancer Center Research Institute, Chiba, Japan; 9 Cancer Prevention and Control Division, Kanagawa Cancer Center Research Institute, Yokohama, Kanagawa Japan; 10 Laboratory of Public Health, School of Food and Nutritional Sciences, University of Shizuoka, Shizuoka, Japan; 11 Nagoya City University Graduate School of Medical Sciences, Nagoya, Aichi Japan; 12 Aichi Cancer Center Research Institute, Nagoya, Aichi Japan; 13 Laboratory for Genotyping Development, RIKEN Center for Integrative Medical Sciences, Yokohama, Kanagawa Japan; 14 Division of Cancer Epidemiology and Prevention, Aichi Cancer Center, Nagoya, Aichi Japan; 15 Division of Descriptive Cancer Epidemiology, Nagoya University Graduate School of Medicine, Nagoya, Aichi Japan; 16 National Institute of Health and Nutrition, National Institutes of Biomedical Innovation, Health and Nutrition, Tokyo, Japan; 17 Department of Preventive Medicine, Nagoya University Graduate School of Medicine, Nagoya, Aichi, Aichi Japan; 18 Division of Cancer Epidemiology, Nagoya University Graduate School of Medicine, Nagoya, Aichi, Japan

**Keywords:** Cancer, Risk factors

## Abstract

The association between vitamin D and total and colorectal cancer risk was inconsistent in observational studies. We conducted Mendelian randomization approach in which the effect of confounding might be reduced. 110 single nucleotide polymorphisms (SNPs) associated with 25-hydroxyvitamin D concentrations were systematically selected according to the “GWAS Catalog” from all ethnic populations. For the SNP-vitamin D concentration association, 3978 individuals from two Japanese cohorts were included. Regarding SNP-total and colorectal cancer association, 4543 cancer cases and 14,224 controls and 7936 colorectal cancer cases and 38,042 controls, respectively were included from the Japanese Consortium of Genetic Epidemiology and other studies in Japan. There was no significant association between the genetically predicted plasma 25-hydroxyvitamin D concentration and total or colorectal cancer in any of the MR analyses. Odds ratios per doubling in vitamin D concentration were 0.83 (95% confidence interval [CI] 0.63–1.09) for total cancer and 1.00 (95% CI 0.80–1.24) for colorectal cancer in inverse variance weighted method, 0.83 (95% CI 0.57–1.19) for total cancer and 1.01 (95% CI 0.75–1.37) for colorectal cancer in MR-Egger method. Consistent with previous MR analyses among European ancestries, there was no significant association identified between 25-hydroxyvitamin D levels and total or colorectal cancer among Asians.

## Introduction

Cancer is a leading cause of death, and research on its preventive factors has been in focus. Dietary factors and related nutrients are modifiable factors, and the associations between these factors and cancer have been investigated.

The association between vitamin D and total and site-specific cancer incidence/mortality has been investigated in many studies, including randomized controlled trials (RCTs), observational studies, and meta-analyses, as vitamin D may play a role in carcinogenesis^1–8^. However, the results of these studies are controversial. A recent meta-analysis that included high-dose vitamin D supplementation RCTs did not find a significant reduction in cancer incidence (Relative risk [RR] 0.98 [95% confidence interval (CI): 0.93– 1.03, *p* = 0.42]^7^, while a meta-analysis of eight prospective studies showed a marginal association between 25(OH)D (a biomarker of vitamin D status in humans) and a lower risk of cancer when the highest with the lowest categories of 25(OH)D were compared (Summary RR = 0.86; 95% CI 0.73–1.02)^8^. An umbrella review of diet and cancer showed that four meta-analyses revealed statistically significant results (*p* < 0.05) among 18 meta-analyses of observational studies on vitamin D and cancer, while heterogeneity was > 50% in nine meta-analyses^9^. Nine out of 18 vitamin D studies showed heterogeneity of > 50%, which was the highest percentage among nutrients included in the umbrella review.

Colorectal cancer is the third most common incident cancer in men and second in women worldwide^10^. This ranking is similar in Japan, and an increasing trend has been observed^11^, while a decline in the incidence of colorectal cancer was found in some countries, including the US^12^. Since the incidence of colorectal cancer is high and anti-tumor effect of vitamin D against colorectal cancer was shown^13,14^, the association between vitamin D concentration and colorectal cancer has been investigated and the present study was conducted under the same hypothesis. However, the previous results remain controversial^15–17^. A meta-analysis showed a significant reduction in colorectal cancer risk in Asians^16^, but not in the latest meta-analysis, which was based on prospective studies^15^. Therefore, in addition to the classical epidemiological method, a new approach is required to investigate the association between vitamin D and cancer.

Mendelian randomization (MR) is an analytical method in which genetic variants are recognized as instrumental variables, and the random allocation of genotypes is likened to randomized trials at conception^18^. Since observational studies have faced controversy regarding the association between vitamin D and cancer, this MR method, in which the effect of confounding factors might be reduced, has been applied to examine these associations. A study from the global network showed that there was not a significant association between genetically determined 25(OH)D and colorectal cancer (the odds ratios [ORs] per 25 nmol/L increment were 0.92 [95% CI 0.76–1.10])^19^, and another study from the UK Biobank also showed that genetically low 25(OH)D levels were not significantly associated with overall cancer risk^20^. Considering the differences in results in a meta-analysis of Asians and ethnic differences in genetic variants, MR studies in Asian populations are required.

To examine the effect of genetically predicted vitamin D concentrations on total and colorectal cancer risk, we conducted an MR analysis in a large-scale case-cohort of 4,543 cancers with a mean follow-up of 15 years among Japanese and 7936 colorectal cancer cases in the Japanese consortium and a combination of several other studies in Japan.

## Results

The basic characteristics of the included studies are presented in Table 1. For the SNP-vitamin D concentration association, 3978 individuals from two Japanese cohorts were included. Regarding SNP-total association, 4543 cancer cases (i.e. individuals with a newly diagnosed cancer) and 14,224 controls were included, and for SNP-colorectal cancer, 7936 colorectal cancer cases and 38,042 controls participated. The mean age of participants the included studies was 52–60 years old. The percentage of men varied among studies and between case and control groups: approximately 35% in the Japan Public Health Centre–based Prospective (JPHC) Study control group and 64% in the BBJ case group. The mean (standard deviation [SD]) of plasma vitamin D was 22.0 (7.2) ng/mL in JPHC and 18.0 (5.1) in the Japan Multi-Institutional Collaborative Cohort (J-MICC) Study.

**Table 1:** Baseline characteristics of participants.

Phenotypes	Study	Source	Total (n)	Men (%)	Age mean (SD)	Crude mean (SD) (ng/mL)
Exposure						
Vitamin D	JPHC-base	Population-based cohort	3739	34.0	53.8 (7.8)	22.0 (7.1)
Vitamin D	J-MICC	Population-based cohort	239	25.1	58.5 (9.7)	18.0 (5.1)

Outcome	Study	Source	Case/Control (n)	Men (%)	Age Mean (SD) among case	Age Mean (SD) among control
Total cancer	JPHC-base	Population-based cohort	3541/10,536	54.1/35.1	56.8 (7.5)	53.9 (7.9)
Total cancer	JPHC-5 year	Population-based cohort	1002/3688	61.1/41.7	55.2 (6.8)	52.9 (7.4)
All (total cancer)			4543/14,224			
Colorectal cancer	JPHC-base	Population-based cohort	482/2434	51.0/35.0	57 (7.3)	53.8 (7.9)
Colorectal cancer	JPHC-5 year	Population-based cohort	194/3607	57.7/41.4	55.5 (6.8)	52.8 (7.4)
Colorectal cancer	J-MICC	Population-based cohort	300/901	62.0/49.4	60.0 (6.5)	55.4 (10.0)
Colorectal cancer	NAGANO	Hhospital-based case–control	105/103	62.9/63.1	59.4 (9.0)	59.2 (8.8)
Colorectal cancer	HERPACC	Hospital-based case–control	163/3819	63.2/49.5	59.4 (10.1)	52.1 (12.2)
Colorectal cancer	BBJ	Hospital-based case–control	6692/27,178	63.7/39.3	66.9 (10.1)	60.7 (10.0)
All (Colorectal cancer)			7936/38,042			

*JPHC* Japan public health center-based prospective, *J-MICC* Japan multi-institutional collaborative cohort, *HERPACC* Hospital-based epidemiologic research program at aichi cancer center, *BBJ* BioBank Japan.

Power calculations under the power of 80% and Type I error rate of 5% were conducted. Minimum detectable ORs per 1 SD increment calculated with the percentage of our explained variance and the number of cases and controls were 0.81 for total cancer and 0.86 for colorectal cancer.

110 single nucleotide polymorphisms (SNPs) were selected from previous studies and associations between SNPs and vitamin D concentrations are shown in Supplemental Table 1. Among the associations between these SNPs and vitamin D in our dataset, two SNPs (rs3755967 and rs10832254) reached genome-wide significance levels (*p*-value < 5.0 × 10^−8^), and 14 SNPs were statistically significant (*p*-value < 0.05) (Table 2). In these significant SNPs, *GC* (chromosome 4, rs3755967) and *CYP24A1* (chromosome 20, rs8121940) which were related to vitamin D metabolism, were detected as nearby genes. D prime values of selected SNPs, rs10832254 (chromosome 11) and rs12803256 (chromosome 11), were both over 0.9 with SNPs near *CYP2R1* and *DHCR7/NADSYN1*, respectively. Regardless of significance or type of nearby gene, we included selected 110 SNPs in MR analyses. The explained variance of 25(OH)D levels by the 110 SNPs was 7.0%. Associations between SNPs and total cancer, or SNPs and colorectal cancer are shown in Supplementary Table 2.

**Table 2:** Summary statistics of the significant SNPs (*p*-value < 0.05) in SNP-exposure association.

SNP	CHR	Position	REF	ALT	Nearby Gene	Beta	SE	*p*_value	Explained variance	F-statistics
rs3755967	4	72,609,398	C	T	*GC*	− 0.10037	0.011	1.78 × 10^−20^	0.0169	86.02529
rs10832254	11	14,434,698	A	G	*COPB1*	− 0.08010	0.009	3.23 × 10^−17^	0.0139	71.20003
rs12803256	11	71,132,868	A	G	*ACTE1P*	0.05003	0.011	1.96 × 10^−06^	0.0045	22.63105
rs2144530	14	39,552,484	C	T	*SEC23A*	− 0.03662	0.010	0.000282	0.0025	13.18342
rs3814995	19	36,342,212	C	T	*NPHS1*	− 0.03342	0.010	0.000605	0.0023	11.75963
rs1229984	4	100,239,319	T	C	*ADH1B*	0.03333	0.011	0.00261945	0.0018	9.05529
rs4575545	16	79,755,446	G	A	*MAFTRR, LINC01229*	− 0.03249	0.011	0.00276995	0.0018	8.95309
rs6724965	2	101,440,151	A	G	*NPAS2*	− 0.02837	0.010	0.00295511	0.0017	8.83494
rs7519574	1	34,726,552	G	A	*C1orf94*	− 0.07541	0.027	0.00514931	0.0017	7.82622
rs8121940	20	52,742,306	C	G	*BCAS1, CYP24A1*	− 0.04614	0.017	0.00648466	0.0015	7.41040
rs2012736	2	234,622,379	C	A	*UGT1A5*	− 0.03686	0.014	0.00838168	0.0014	6.95009
rs8091117	18	28,919,794	C	A	*DSG1*	− 0.05525	0.022	0.0134677	0.0012	6.10659
rs35823191	1	17,560,123	T	C	*PADI1*	− 0.02786	0.013	0.0320312	0.0009	4.59680
rs11732896	4	88,287,993	G	A	*HSD17B11*	− 0.02169	0.010	0.0344214	0.0009	4.47363

*Chr* Chromosome position: Chromosomal Position (hg19), *REF* Reference allele, *ALT* Alternative allele, *SE* Standard error, Approximate F-statistics which is calculated from the formula (beta/se)^2^.

**Figure 1:**
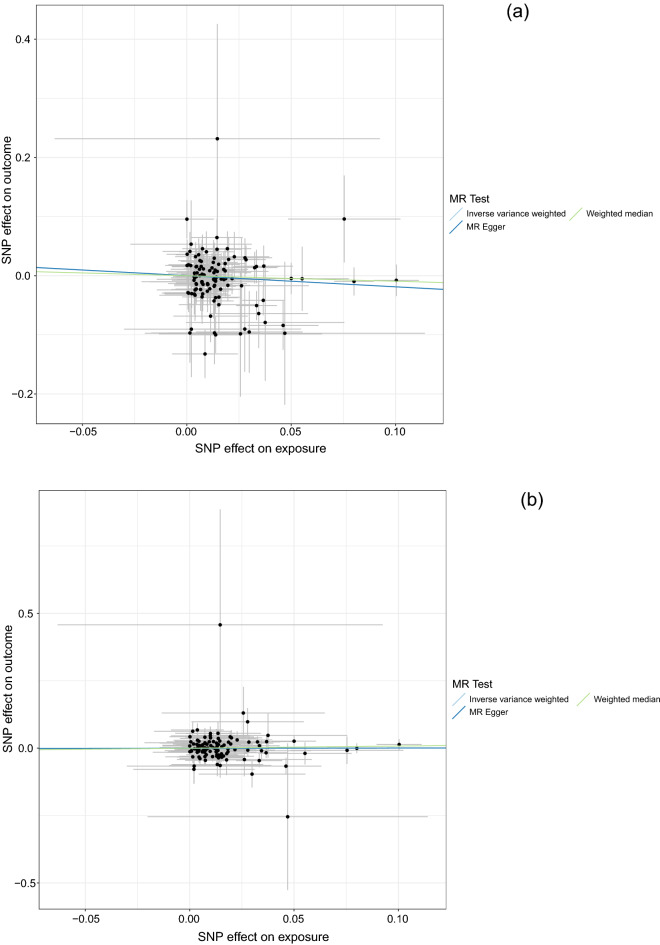
Analyses of vitamin D and total cancer risk (**a**) and colorectal cancer risk (**b**) with 110 SNPs.

The MR results between vitamin D and total cancer or colorectal cancer are shown in Table 3, and their scatter plots with 110 SNPs are shown in Fig. 1. There were no significant associations between genetically predicted plasma vitamin D levels and total or colorectal cancer in any of the MR methods. ORs per 1 unit increase in log_2_-transformed vitamin D concentration (95% CI) were 0.83 (0.63–1.09) for total cancer and 1.00 (0.80–1.24) for colorectal cancer in random-effect inverse-variance weighted (IVW) method. Results were not significant in MR-Egger method (0.83 [0.57–1.19] for total cancer and 1.01 [0.75–1.37] for colorectal cancer) and weighted median method (0.91 [0.62–1.34] for total cancer and 1.08 [0.79–1.48] for colorectal cancer). When we only included significant 14 SNPs in Table 2, MR results were not significant similarly. MR-Egger intercepts were not significant in either the total cancer or colorectal cancer models. Since p-value of Q statistics was not significant for total cancer but marginal (*p* = 0.05) for colorectal cancer in the heterogeneity test, the random-effect IVW method was used. MR-PRESSO method was used to detect horizontal pleiotropic outliers, however, there were no outliers for total and colorectal cancer assessment. No single SNP changed the result according to the leave-one-out analysis. When we included strong instruments (*p*-value < 5 × 10^−6^ or approximate F-statistics > 10) only in MR analysis to avoid weak instrument bias, heterogeneity became non-significant, however, non-significant results were not changed (Supplemental Table 3). Moreover, JPHC-base sample for SNP-exposure association overlapped with JPHC-base in SNP-total and colorectal cancer associations. We conducted sensitivity analysis excluding JPHC-base samples from SNP-outcome association and perform MR analysis. The results for total cancer and colorectal cancer were not changed largely and remained non-significant (Supplemental Table 4).

**Table 3:** Mendelian Randomization estimates between plasma vitamin D concentrations and total or colorectal cancer.

MR method	Odds ratio (95% CI) with 110 SNPs	*p* value	Odds ratio (95% CI) with 14 SNPs^a^	*p* value
Total cancer				
IVW	0.83 (0.63–1.09)	0.18	0.88 (0.64–1.22)	0.45
MR Egger	0.83 (0.57–1.19)	0.31	0.91 (0.43–1.90)	0.80
Egger intercept	–	0.96	–	0.93
Weighted median	0.91 (0.62–1.34)	0.63	0.91 (0.62–1.35)	0.64
Colorectal cancer				
IVW	1.00 (0.80–1.24)	0.98	1.05 (0.82–1.34)	0.71
MR Egger	1.01 (0.75–1.37)	0.93	1.09 (0.62–1.90)	0.77
Egger intercept	–	0.88	–	0.88
Weighted median	1.08 (0.79–1.48)	0.62	1.09 (0.80–1.47)	0.60

*IVW* Inverse variance weighted, *MR Egger* Mendelian randomization egger.

ORs were per 1 unit increment in log_2_-transformed Vitamin D concentrations.

^a^14 SNPSs (*p* < 0.05) were rs3755967, rs10832254, rs12803256, rs2144530, rs3814995,rs1229984, rs4575545, rs6724965, rs7519574, rs8121940, rs2012736, rs8091117, rs35823191, and rs11732896 in Table 2.

## Discussion

In an MR analysis using a meta-analysis of large cohorts or case–control studies with genetic information in Japan, no significant association between genetically predicted plasma vitamin D and total or colorectal cancer was found. Although an observational study from a Japanese cohort showed a significant association between plasma vitamin D levels and a lower risk of total cancer^4^, we did not find a significant association between vitamin D and total cancer or colorectal cancer through the MR framework.

Although controversial results have been proposed in observational studies and some previous MR studies focused on vitamin D and cancer^1–8,19–21^, the primary reason for conducting this MR analysis is that vitamin D concentration was significantly associated with a lower risk of total cancer in an observational study in Japan^4^. Our null findings were, however, consistent with a previous MR study of European ancestries^20^, although we focused on Asian populations who may have a different association because of their different genetic backgrounds. Regarding overall cancer incidence, Ong et al. showed non-significant results (combined OR [95% CI] 0.97 [0.90–1.04]) from the UK biobank, including 46,155 cancer cases^22^. Although this study used six SNPs that explained 3.5% of the variation in vitamin D concentration, the authors conducted a reassessment for various types of cancer using 74 SNPs, which explained up to 4% of the variation. Nevertheless, they did not find a significant association between most types of cancer other than ovarian cancer^21^. Although we selected 110 SNPs, these were based on studies mainly from European ancestries and no study for East Asian only genome-wide association study (GWAS) was published so far. Because of this, a few instrumental variables were significantly associated with Vitamin D concentration. Explained variance was 7.0% in our samples, however, both analyses with all 110 SNPs and with significant SNPs only showed null associations. Considering the null results for most types of cancer in the large MR studies^21^, our null findings for total cancer were agreeable, although we included a relatively small sample size compared with European ancestry studies, which might be judged from the sample size calculation result.

Regarding colorectal cancer, MR analysis from the large consortium with 11,488 colorectal cancer cases showed that the OR was 0.92 (95% CI 0.76–1.10)^19^ and bidirectional MR analysis with 26,397 cases also did not show significant results for vitamin D and colorectal cancer risk^23^. Our results are consistent with these findings. Among observational studies, a study in Japan that showed a significant reduction in overall cancer showed null results in the case of colorectal cancer (OR in Q4 vs. Q1: 0.95 [95% CI 0.73–1.23], *p* for trend = 0.48)^4^. Inconsistent with this, a pooled analysis of 17 cohorts showed a significant reduction in the high vitamin D concentration (87.5– < 100 nmol/L) group^24^ and the meta-analyses from Asians showed significant dose–response reduction^14^. We observed a discrepancy between MR analysis and observational studies among Asians. A potential reason for this might be unknown or unmeasured confounders in the models used in observational studies or because our results were from a linear MR Further MR studies are required to confirm these results.

In vitro studies have shown that high concentrations of vitamin D inhibit tumor cell proliferation and induce differentiation^13^. The anti-tumor effects of vitamin D include pro-apoptosis, anti-proliferation, and pro-differentiation effects^25^. Based on these mechanisms of antitumor effects, epidemiological studies have shown the potential of vitamin D in cancer prevention. Genetic variations in vitamin D status, however, have not shown significant results, similar to those of MR studies^26,27^. As with our results, SNPs detected in GWAS studies explained a small percentage of vitamin D concentrations, and it might be difficult to detect the reduction in risk of cancer caused by the increased genetically predicted vitamin D levels. Other potential reasons that we did not find significant results were as follows: (i) the causal relationship between vitamin D level and cancer did not truly exist, and (ii) the power was was limited to detect an existing, but small effect. Because of the relatively low explained variance of vitamin D, GWAS of Vitamin D including more Japanese participants is required to allow MR with a larger sample size, and large randomized controlled trials to investigate the causal relationship between vitamin D and cancer in Japanese are needed.

A strength of this study is the application of a two-sample MR framework in a relatively large-scale Asian population to examine the association between vitamin D and cancer. The MR method can overcome the potential bias in observational findings. However, our study has few limitations. First, we assumed a linear association between vitamin D and colorectal cancer, and we could not investigate the nonlinear effect because we could not include individual-level data. Second, the sample size was small. In SNP exposure analyses, vitamin D was measured in a limited number of cohort studies because measurement of vitamin D concentration in many samples was not feasible in prospective cohorts. Based on the sample size calculation, a relatively weak association may not be detectable in this sample size. In addition, the measurement was conducted only once, and measurement errors could not be excluded. In the SNP outcome analysis, site-specific cancers other than colorectal cancer were not included because of the limited number of cases. Third, the selected SNPs were based on previously published studies that did not include the East Asian population. For this reason, we included both significant and non-significant instrumental variables in MR and this may cause weak instrument bias. Although sensitivity analysis with significant instruments showed similar results with main analyses, GWAS results for vitamin D in a Japanese population are required. Also, this study was conducted in Japan, so generalizability might be limited to Japanese. Fourth, because we could not collect individual genetic data and used summary results of GWAS as estimates, we did not assess the association of colorectal cancer by site. However, since the association between vitamin D concentration and cancer is controversial, and evidence from the Asian population is scarce, this study is worth reporting. Further studies with a larger sample size are required to confirm this hypothesis.

In conclusion, consistent with MR studies in European ancestries, there was no statistically significant association between vitamin D concentrations and total and colorectal cancer risk from MR analysis among the Japanese population.

## Methods

We performed a two-sample MR analysis in which two types of estimates from two separate datasets were used to evaluate the objective association (vitamin D concentration and total or colorectal cancer in this study). One is the estimate between SNPs and the exposure of the objective association (i.e., vitamin D in this study), and the other is the estimate between SNP and the outcome (total or colorectal cancer). MR analysis should be based on the following three assumptions: (i) SNPs (as instrumental variables) are associated with the exposure, (ii) SNPs (as instrumental variables) are not associated with confounding factors that are supposed to exist in the association between the exposure and the outcome, and (iii) SNPs as instrumental variables are not directly associated with the outcome and are related to the outcome only through exposure^18,22^.

### Methods for selecting SNPs associated with vitamin D

SNPs used as instrumental variables in the MR analysis were selected according to previously published papers. We used the National Human Genome Research Institute-European Bioinformatics Institute (NHGRI-EBI) database named GWAS Catalog (https://www.ebi.ac.uk/gwas/) to select SNPs. In September 2022, 713 SNPs, including duplicates, were shown to be associated with “vitamin D measurements” in the GWAS Catalog. We systematically chose SNPs for instrumental variables in the following criteria; (a) phenotype was vitamin D measurement (647 SNPs remained), (b) *p*-value of SNPs were < 5 × 10^−8^ (614 SNPs remained) (c) participants in the original papers were adults (609 SNPs remained), (d) duplicate of SNPs (475 SNPs remained), (e) minor allele frequency (MAF) > 0.01 in the East Asian populations based on 1000 genome project (325 SNPs remained), (f) clumping using “clump_data” (clumping 10,000 kb window and R^2^ > 0.001) in “TwosampleMR (version 0.5.6)” library of R software. Finally, 110 SNPs were used for this analysis. (Supplemental Table 1).

### The data source of MR analysis in Japanese

We calculated the estimates of SNP-vitamin D association from two Japanese cohorts: JPHC Study and J-MICC Study. The details of each study are provided in Supplemental Table 5. Outliers of vitamin D measurement (70 ng/mL) were excluded, and vitamin D was log_2_ transformed to be close to a normal distribution. Estimates were calculated using a linear regression model among 3739 JPHC participants and 239 J-MICC participants. The models were adjusted for age, sex, season, PCA, and area (JPHC only). A meta-analysis was performed for each target SNP using a fixed effects model.

For the SNP-outcome association, we examined two types of cancer (total and colorectal cancer). We selected colorectal cancer as a site-specific cancer because it is one of the most common cancers, and we could include a sufficient sample size of cancer cases. For total cancer, 3541 cases and 10,536 controls were identified from the participants who answered the baseline questionnaire of the JPHC (JPHC-base) and provided blood samples. A total of 1002 cases and 3688 controls were identified from participants who answered the 5-year questionnaire of JPHC, provided blood samples, and were not included in the baseline analysis (JPHC-5 year). For colorectal cancer, colorectal cancer cases and controls from the JPHC Study, NAGANO Study, Hospital-based Epidemiologic Research Program at Aichi Cancer Centre (HERPACC Study), J-MICC Study, and non-restricted published genome-wide association study (GWAS) analysis data from BioBank Japan (BBJ) were gathered and meta-analyzed for target SNPs. The descriptions of these studies are shown in Supplemental Table 5, and the genotyping, imputation method, and details of the association studies in each study are described in Supplemental Table 6.

We performed power calculations with mRnd (https://shiny.cnsgenomics.com/mRnd) and a type-I error rate of 5% and power of 80% were set.

This study was approved by the review board of the National Cancer Center, Japan, and all the participating studies were approved by each institutional review boards and informed consent was obtained from all participants. All methods were performed in accordance with the Declaration of Helsinki and Japanese ethical guidelines. Details are shown in Supplemental Table 5. Our study protocol was approved among researchers in participated studies before analyses and this study is reported following the “Strengthening the Reporting of Observational Studies in Epidemiology Using Mendelian Randomization” (STROBE-MR)^28^. Statement in Supplemental Table 7.

### Statistical analysis

In this MR analysis, estimates (β coefficients and 95% CI) of selected SNPs from previous studies were calculated in our dataset (both SNP-exposure and SNP-outcome associations) and used in MR analysis to avoid overestimation of the association (called the Beavis effect, or the winner’s curse)^29,30^. Estimates and other related information on targeted SNPs were collected and used for calculation. In SNP-exposure analysis, explained variance and F-statistics for each SNP were calculated. Approximate F-statistics were calculated from the formula (beta/standard error)^2^. After calculating the β coefficients for SNP-exposure and SNP-outcome associations in each study, we combined them with IVW in a fixed or random-effect model depending on the heterogeneity test. 110 SNPs identified from previous studies were regarded as instrumental variables. We selected them irrespective of the statistical significance of these SNPs in our data because overfitting or insufficient power in our data might cause bias in SNP selection.

We performed MR analysis with 110 SNPs and significant (*p*-value < 0.05) 14 SNPs using “TwoSampleMR” (version 0.5.6) available as R package. All these procedures were conducted using the Statistical software R version 3.5.0 (R Foundation for Statistical Computing, Vienna, Austria). To examine the association, the IVW, MR-Egger regression, and the weighted-median method were used. Because the IVW method is likely to be affected by horizontal pleiotropy, MR-Egger regression, in which the intercept reflects the pleiotropic condition, was conducted and MR-PRESSO method was used to detect and exclude horizontal pleiotropic outliers. The weighted-median method was used because consistent estimates were obtained from the weighted-median method when invalid instrumental variables were included. As sensitivity analysis, significant (*p* < 5 × 10^−6^) or strong (F-statistics > 10) instruments were selected and included in MR analysis. Moreover, we further conducted MR analysis without the result from JPHC base in SNP-outcome analysis to avoid sample overlapping. The significance of the association with MR was set at *p *< 0.05.

## Supplementary Information

Supplementary Tables.

## Data Availability

SNP data analysed in the current study are available in the NHGRI-EBI GWAS Catalog repository, https://www.ebi.ac.uk/gwas/.

## References

[1] Lappe J (2017). Effect of vitamin D and calcium supplementation on cancer incidence in older women: A randomized clinical trial. JAMA.

[2] Scragg R (2018). Monthly high-dose vitamin D supplementation and cancer risk: A post hoc analysis of the vitamin D assessment randomized clinical trial. JAMA Oncol..

[3] Manson JE (2019). Vitamin D supplements and prevention of cancer and cardiovascular disease. N. Engl. J. Med..

[4] Budhathoki S (2018). Plasma 25-hydroxyvitamin D concentration and subsequent risk of total and site specific cancers in Japanese population: Large case-cohort study within Japan public health center-based prospective study cohort. BMJ.

[5] Ordóñez-Mena JM (2013). Serum 25-hydroxyvitamin D and cancer risk in older adults: results from a large German prospective cohort study. Cancer Epidemiol. Biomarkers Prev..

[6] de Boer IH (2012). Serum 25-hydroxyvitamin D concentration and risk for major clinical disease events in a community-based population of older adults a cohort study. Ann. Intern. Med..

[7] Keum N, Lee DH, Greenwood DC, Manson JE, Giovannucci E (2019). Vitamin D supplementation and total cancer incidence and mortality: A meta-analysis of randomized controlled trials. Ann. Oncol..

[8] Han J (2019). 25-Hydroxyvitamin D and total cancer incidence and mortality: A meta-analysis of prospective cohort studies. Nutrients.

[9] Papadimitriou N (2021). An umbrella review of the evidence associating diet and cancer risk at 11 anatomical sites. Nat. Commun..

[10] Sung H (2021). Global cancer statistics 2020: GLOBOCAN estimates of incidence and mortality worldwide for 36 cancers in 185 countries. CA Cancer J. Clin..

[11] National Cancer Center Japan. Statistics in Cancer Information Service. https://ganjoho.jp/reg_stat/statistics/stat/cancer/67_colorectal.html#anchor1, (2021).

[12] Arnold M (2020). Global burden of 5 major types of gastrointestinal cancer. Gastroenterology.

[13] Samuel S, Sitrin MD (2008). Vitamin D's role in cell proliferation and differentiation. Nutr. Rev..

[14] Ferrer-Mayorga G, Larriba MJ, Crespo P, Muñoz A (2019). Mechanisms of action of vitamin D in colon cancer. J. Steroid Biochem. Mol. Biol..

[15] Boughanem H (2021). Vitamin D intake and the risk of colorectal cancer: An updated meta-analysis and systematic review of case-control and prospective cohort studies. Cancers (Basel).

[16] Zhang L (2019). Association between blood circulating vitamin D and colorectal cancer risk in Asian countries: A systematic review and dose-response meta-analysis. BMJ (Open).

[17] Ma Y (2011). Association between vitamin D and risk of colorectal cancer: A systematic review of prospective studies. J. Clin. Oncol..

[18] Davies NM, Holmes MV, Davey Smith GV (2018). Reading Mendelian randomisation studies: A guide, glossary, and checklist for clinicians. BMJ.

[19] Dimitrakopoulou VI (2017). Circulating vitamin D concentration and risk of seven cancers: Mendelian randomisation study. BMJ.

[20] Ong JS (2018). Vitamin D and overall cancer risk and cancer mortality: a Mendelian randomization study. Hum. Mol. Genet..

[21] Ong JS (2021). A comprehensive re-assessment of the association between vitamin D and cancer susceptibility using Mendelian randomization. Nat. Commun..

[22] Didelez V, Sheehan N (2007). Mendelian randomization as an instrumental variable approach to causal inference. Stat. Methods Med. Res..

[23] He Y (2022). Bidirectional Mendelian randomisation analysis of the relationship between circulating vitamin D concentration and colorectal cancer risk. Int. J. Cancer.

[24] McCullough ML (2019). Circulating vitamin D and colorectal cancer risk: an international pooling project of 17 cohorts. J. Natl. Cancer Inst..

[25] Jeon SM, Shin EA (2018). Exploring vitamin D metabolism and function in cancer. Exp. Mol. Med..

[26] Hiraki LT (2013). Genetic predictors of circulating 25-hydroxyvitamin d and risk of colorectal cancer. Cancer Epidemiol. Biomark. Prev..

[27] Jorde R (2012). Polymorphisms related to the serum 25-hydroxyvitamin D level and risk of myocardial infarction, diabetes, cancer and mortality. The Tromsø study. PLOS ONE.

[28] Skrivankova VW (2021). Strengthening the reporting of observational studies in epidemiology using mendelian randomization (STROBE-MR) statement. JAMA.

[29] Taylor AE (2014). Mendelian randomization in health research: Using appropriate genetic variants and avoiding biased estimates. Econ. Hum. Biol..

[30] Haycock PC (2016). Best (but oft-forgotten) practices: The design, analysis, and interpretation of Mendelian randomization studies. Am. J. Clin. Nutr..

