# Supplementary material for: Association of 25-hydroxyvitamin D with risk of overall and colorectal cancer among Japanese using a Mendelian randomization approach
Source: Sci Rep. 2023 Feb 10;13:2384. doi: 10.1038/s41598-023-29596-8 (PMC9918529; doi:10.1038/s41598-023-29596-8)
Supplement: Supplementary file 1 — Supplementary Tables. [file 41598_2023_29596_MOESM1_ESM.pdf]

Title: Association of 25-hydroxyvitamin D with risk of overall and colorectal cancer among Japanese using Mendelian randomization approach

Authors: Ryoko Katagiri, Atsushi Goto, Shiori Nakano, Masahiro Nakatochi, Yuriko N. Koyanagi, Masao Iwagami, Akiko Hanyuda, Taiki Yamaji, Norie Sawada, Yohko Nakamura, Sho Nakamura, Kiyonori Kuriki, Sadao Suzuki, Issei Imoto, Yukihide Momozawa, Isao Oze, Hidemi Ito, Shoichiro Tsugane, Kenji Wakai, Keitaro Matsuo and Motoki Iwasaki

Supplemental Table 1. **Summary statistics of the selected 110 SNPs in SNP-exposure (Vitamin D) association**

SNP ID	Chr	Position	REF	ALT	EAF	Beta	SE	P_value	OR	UCL	LCL	Explained variance	F-statistics
rs903911	1	2326812	A	G	0.28	0.0045	0.01	0.6763	1.00	1.03	0.98	0.000035	0.17
rs35823191	1	17560123	T	C	0.16	-0.0279	0.01	0.0320	0.97	1.00	0.95	0.000905	4.60
rs7519574	1	34726552	G	A	0.04	-0.0754	0.03	0.0051	0.93	0.98	0.88	0.001668	7.83
rs1343776	1	41757718	G	A	0.33	-0.0098	0.01	0.3366	0.99	1.01	0.97	0.000183	0.92
rs10789481	1	46383583	C	G	0.62	0.0045	0.01	0.6479	1.00	1.02	0.99	0.000042	0.21
rs2131925	1	63025942	G	T	0.78	-0.0006	0.01	0.9616	1.00	1.02	0.98	0.000000	0.00
rs7528419	1	109817192	A	G	0.07	0.0099	0.02	0.5997	1.01	1.05	0.97	0.000054	0.28
rs9050	1	152079314	C	A	0.02	0.0375	0.04	0.3224	1.04	1.12	0.96	0.000270	0.98
rs576242124	1	152390763	G	A	0.01	0.0146	0.08	0.8513	1.01	1.18	0.87	0.000013	0.04
rs12144049	1	152440910	C	T	0.76	-0.0186	0.01	0.1213	0.98	1.00	0.96	0.000553	2.40
rs2807834	1	220970593	T	G	0.84	-0.0094	0.01	0.4741	0.99	1.02	0.97	0.000100	0.51
rs4846913	1	230294715	C	A	0.19	0.0138	0.01	0.2479	1.01	1.04	0.99	0.000257	1.34
rs1260326	2	27730940	T	C	0.45	-0.0110	0.01	0.2500	0.99	1.01	0.97	0.000259	1.32
rs35270497	2	38259872	C	T	0.14	0.0078	0.01	0.5730	1.01	1.04	0.98	0.000064	0.32
rs727857	2	58981967	G	A	0.44	0.0178	0.01	0.0649	1.02	1.04	1.00	0.000674	3.41
rs2710651	2	63166379	G	A	0.54	0.0069	0.01	0.4757	1.01	1.03	0.99	0.000103	0.51
rs6724965	2	101440151	A	G	0.48	-0.0284	0.01	0.0030	0.97	0.99	0.95	0.001745	8.83
rs7569755	2	118648261	G	A	0.09	0.0228	0.02	0.1755	1.02	1.06	0.99	0.000374	1.84
rs1047891	2	211540507	C	A	0.14	-0.0036	0.01	0.8007	1.00	1.02	0.97	0.000013	0.06
rs2012736	2	234622379	C	A	0.14	-0.0369	0.01	0.0084	0.96	0.99	0.94	0.001382	6.95
rs9847248	3	18804655	G	A	0.63	0.0001	0.01	0.9885	1.00	1.02	0.98	0.000000	0.00
rs1128535	3	49866392	C	T	0.22	-0.0037	0.01	0.7448	1.00	1.02	0.97	0.000021	0.11
rs7652808	3	85603643	T	G	0.93	0.0154	0.02	0.4232	1.02	1.05	0.98	0.000130	0.64
rs6438900	3	125148287	C	G	0.18	-0.0144	0.01	0.2443	0.99	1.01	0.96	0.000265	1.36
rs34186890	3	141720712	A	G	0.30	0.0063	0.01	0.5461	1.01	1.03	0.99	0.000072	0.36
rs1949633	3	153758806	T	C	0.67	-0.0069	0.01	0.4898	0.99	1.01	0.97	0.000092	0.48
rs78649910	4	3482213	T	A	0.31	0.0001	0.01	0.9912	1.00	1.02	0.98	0.000000	0.00
rs4364259	4	15892159	G	A	0.19	0.0083	0.01	0.5435	1.01	1.04	0.98	0.000093	0.37
rs4616820	4	57745481	C	T	0.54	0.0130	0.01	0.1668	1.01	1.03	0.99	0.000365	1.91
rs7665631	4	70281414	A	C	0.18	-0.0037	0.01	0.7721	1.00	1.02	0.97	0.000017	0.08
rs3755967	4	72609398	C	T	0.26	-0.1004	0.01	0.0000	0.90	0.92	0.89	0.016886	86.03
rs10021949	4	72764039	A	G	0.81	0.0015	0.01	0.9101	1.00	1.03	0.98	0.000003	0.01

rs11732896	4	88287993	G	A	0.30	-0.0217	0.01	0.0344	0.98	1.00	0.96		0.000858	4.47
rs1229984	4	100239319	T	C	0.25	0.0333	0.01	0.0026	1.03	1.06	1.01		0.001821	9.06
rs7718395	5	118652574	C	G	0.02	0.0262	0.03	0.4170	1.03	1.09	0.96		0.000133	0.66
rs7712001	5	148020950	T	G	0.56	0.0130	0.01	0.1831	1.01	1.03	0.99		0.000363	1.77
rs79666294	5	154426706	C	T	0.02	-0.0021	0.03	0.9469	1.00	1.06	0.94		0.000001	0.00
rs1321247	6	25662873	A	T	0.03	0.0128	0.03	0.6373	1.01	1.07	0.96		0.000044	0.22
rs742493	6	40998167	T	C	0.05	-0.0132	0.02	0.5465	0.99	1.03	0.95		0.000072	0.36
rs9476310	6	57767576	C	T	0.42	-0.0044	0.01	0.7000	1.00	1.02	0.97		0.000042	0.15
rs12153819	6	83773049	C	T	0.14	-0.0061	0.01	0.6599	0.99	1.02	0.97		0.000038	0.19
rs9375037	6	121856794	A	C	0.34	0.0121	0.01	0.2268	1.01	1.03	0.99		0.000283	1.46
rs2248551	6	131924689	G	A	0.33	0.0152	0.01	0.1344	1.02	1.04	1.00		0.000448	2.24
rs10085881	7	21577960	T	C	0.14	0.0066	0.01	0.6402	1.01	1.03	0.98		0.000046	0.22
rs2595644	7	43980540	G	T	0.56	-0.0075	0.01	0.4373	0.99	1.01	0.97		0.000121	0.60
rs7784802	7	64015379	A	T	0.20	0.0145	0.01	0.2254	1.01	1.04	0.99		0.000296	1.47
rs41301394	7	75612803	C	T	0.40	0.0062	0.01	0.5255	1.01	1.03	0.99		0.000081	0.40
rs1011468	7	104613791	G	A	0.94	0.0136	0.02	0.5075	1.01	1.06	0.97		0.000092	0.44
rs1858889	7	107117447	A	C	0.41	0.0073	0.01	0.4669	1.01	1.03	0.99		0.000112	0.53
rs2346264	7	133536351	A	C	0.96	-0.0072	0.02	0.7732	0.99	1.04	0.95		0.000017	0.08
rs4841132	8	9183596	A	G	0.96	0.0257	0.04	0.5107	1.03	1.11	0.95		0.000245	0.43
rs804281	8	11611865	A	G	0.97	0.0020	0.03	0.9440	1.00	1.06	0.95		0.000001	0.00
rs28692966	8	25892919	G	A	0.38	-0.0045	0.01	0.6483	1.00	1.01	0.98		0.000041	0.21
rs4738684	8	59393273	A	G	0.82	0.0009	0.01	0.9470	1.00	1.03	0.98		0.000001	0.00
rs12056768	8	116988527	T	G	0.52	-0.0101	0.01	0.2867	0.99	1.01	0.97		0.000222	1.14
rs13294734	9	80710910	C	T	0.26	0.0057	0.01	0.6049	1.01	1.03	0.98		0.000054	0.27
rs13284054	9	107669073	T	C	0.07	-0.0040	0.02	0.8296	1.00	1.03	0.96		0.000009	0.05
rs10818769	9	125719923	C	G	0.21	-0.0194	0.01	0.0893	0.98	1.00	0.96		0.000553	2.89
rs532436	9	136149830	G	A	0.26	-0.0138	0.01	0.2012	0.99	1.01	0.97		0.000321	1.63
rs2398113	10	10076429	A	G	0.38	0.0162	0.01	0.1016	1.02	1.04	1.00		0.000534	2.68
rs10887718	10	82042624	C	T	0.92	0.0165	0.02	0.3451	1.02	1.05	0.98		0.000173	0.89
rs12775091	10	91524012	C	T	0.36	0.0049	0.01	0.6170	1.00	1.02	0.99		0.000048	0.25
rs2297991	10	113913222	T	C	0.80	0.0047	0.01	0.6940	1.00	1.03	0.98		0.000031	0.15
rs2618515	11	14012957	A	G	0.70	-0.0131	0.01	0.2040	0.99	1.01	0.97		0.000318	1.61
rs10832254	11	14434698	A	G	0.50	-0.0801	0.01	0.0000	0.92	0.94	0.91		0.013923	71.20
rs71473824	11	71063989	C	T	0.24	-0.0099	0.01	0.3952	0.99	1.01	0.97		0.000158	0.72
rs12803256	11	71132868	A	G	0.30	0.0500	0.01	0.0000	1.05	1.07	1.03		0.004521	22.63
rs964184	11	116648917	G	C	0.70	0.0131	0.01	0.2126	1.01	1.03	0.99		0.000312	1.55
rs2847500	11	120114421	G	A	0.09	0.0344	0.02	0.1456	1.04	1.08	0.99		0.000822	2.12

rs1871395	12	21352315	A	G	0.34	-0.0154	0.01	0.1316	0.98	1.00	0.97	0.000462	2.27
rs1396206	12	24576859	A	T	0.97	-0.0073	0.03	0.8010	0.99	1.05	0.94	0.000012	0.06
rs11182428	12	38526387	T	C	0.20	0.0073	0.01	0.5616	1.01	1.03	0.98	0.000074	0.34
rs1038165	12	68665940	C	T	0.43	0.0008	0.01	0.9328	1.00	1.02	0.98	0.000001	0.01
rs10859995	12	96375682	T	C	0.41	-0.0100	0.01	0.3006	0.99	1.01	0.97	0.000210	1.07
rs73413596	12	111582630	T	C	0.03	0.0278	0.03	0.3025	1.03	1.08	0.98	0.000220	1.06
rs28435470	12	133067473	G	A	0.12	-0.0200	0.01	0.1721	0.98	1.01	0.95	0.000371	1.86
rs4580037	13	55702646	A	C	0.50	0.0046	0.01	0.6333	1.00	1.02	0.99	0.000045	0.23
rs2144530	14	39552484	C	T	0.68	-0.0366	0.01	0.0003	0.96	0.98	0.95	0.002523	13.18
rs2756119	14	104001517	G	A	0.55	0.0019	0.01	0.8498	1.00	1.02	0.98	0.000007	0.04
rs1800588	15	58723675	C	T	0.51	0.0073	0.01	0.4441	1.01	1.03	0.99	0.000115	0.59
rs62007299	15	77711719	G	A	0.39	0.0042	0.01	0.6697	1.00	1.02	0.99	0.000036	0.18
rs325384	15	100229761	C	T	0.29	-0.0034	0.01	0.7464	1.00	1.02	0.98	0.000021	0.10
rs1684600	16	4594671	C	T	0.30	0.0197	0.01	0.0658	1.02	1.04	1.00	0.000698	3.38
rs8063706	16	11909552	A	T	0.38	0.0127	0.01	0.2773	1.01	1.04	0.99	0.000327	1.18
rs77924615	16	20392332	G	A	0.22	0.0142	0.01	0.2290	1.01	1.04	0.99	0.000295	1.45
rs71383766	16	30930233	C	T	0.89	0.0087	0.02	0.5799	1.01	1.04	0.98	0.000064	0.31
rs11076175	16	57006378	A	G	0.08	-0.0114	0.02	0.5240	0.99	1.02	0.95	0.000082	0.41
rs4575545	16	79755446	G	A	0.27	-0.0325	0.01	0.0028	0.97	0.99	0.95	0.001805	8.95
rs10454087	17	40735641	C	T	0.48	-0.0135	0.01	0.1479	0.99	1.00	0.97	0.000398	2.09
rs2952289	17	66464414	C	T	0.61	0.0184	0.01	0.0606	1.02	1.04	1.00	0.000695	3.52
rs61698755	17	79257880	T	C	0.35	-0.0035	0.01	0.7297	1.00	1.02	0.98	0.000024	0.12
rs8091117	18	28919794	C	A	0.05	-0.0552	0.02	0.0135	0.95	0.99	0.91	0.001202	6.11
rs4121823	18	47144223	T	A	0.79	0.0076	0.01	0.5162	1.01	1.03	0.98	0.000084	0.42
rs2037511	18	61366207	G	A	0.16	0.0000	0.01	0.9977	1.00	1.03	0.97	0.000000	0.00
rs57631352	19	4338173	A	G	0.45	-0.0079	0.01	0.4058	0.99	1.01	0.97	0.000136	0.69
rs142158911	19	11190534	G	A	0.01	-0.0469	0.07	0.4843	0.95	1.09	0.84	0.000211	0.49
rs12462826	19	11955767	G	A	0.04	0.0299	0.03	0.2422	1.03	1.08	0.98	0.000265	1.37
rs58542926	19	19379549	C	T	0.08	0.0130	0.02	0.4663	1.01	1.05	0.98	0.000104	0.53
rs3814995	19	36342212	C	T	0.62	-0.0334	0.01	0.0006	0.97	0.99	0.95	0.002278	11.76
rs7412	19	45412079	C	T	0.04	0.0146	0.02	0.5512	1.01	1.06	0.97	0.000070	0.36
rs62130059	19	48461240	C	A	0.75	0.0177	0.01	0.1283	1.02	1.04	0.99	0.000510	2.31
rs10426	19	51517798	G	A	0.07	0.0137	0.02	0.4549	1.01	1.05	0.98	0.000112	0.56
rs35313547	19	58352806	T	C	0.11	-0.0039	0.02	0.8184	1.00	1.03	0.96	0.000014	0.05
rs2207173	20	22805061	G	A	0.59	-0.0023	0.01	0.8097	1.00	1.02	0.98	0.000011	0.06
rs6129648	20	39231118	A	G	0.55	-0.0183	0.01	0.0549	0.98	1.00	0.96	0.000722	3.69
rs158528	20	52721956	A	G	0.46	-0.0138	0.01	0.1489	0.99	1.00	0.97	0.000409	2.08

rs2585442	20	52737123	C	G	0.07	0.0015	0.02	0.9369	1.00	1.04	0.97	0.000001	0.01
rs8121940	20	52742306	C	G	0.09	-0.0461	0.02	0.0065	0.95	0.99	0.92	0.001479	7.41
rs2074735	22	31535872	G	C	0.34	0.0110	0.01	0.2813	1.01	1.03	0.99	0.000238	1.16
rs138335	22	41227086	C	G	0.44	-0.0133	0.01	0.1670	0.99	1.01	0.97	0.000380	1.91

Chr: Chromosome Position: Chromosomal Position (hg19) REF: Reference allele ALT: Alternative allele EAF: Effect allele frequency. SE: standard error OR: odds ratio UC, LCL: Upper and lower confidence limit of 95% confidence interval

Total explained variance: 6.7%

Approximate F-statistics which is calculated from the formula $(beta/se)^2$

Supplemental Table 2. **Summary statistics of the selected SNPs in SNP-outcome (Total and colorectal cancer) associations**

					Total Cancer						Colorectal Cancer					
SNP ID	Chr	Position	REF	ALT	Beta	SE	P_value	OR	LCL	UCL	Beta	SE	P_value	OR	LCL	UCL
rs903911	1	2326812	A	G	-0.0134	0.03	0.6132	0.99	0.94	1.04	-0.03284	0.02	0.1067	0.97	0.93	1.01
rs35823191	1	17560123	T	C	-0.0303	0.03	0.3584	0.97	0.91	1.03	0.00729	0.02	0.7692	1.01	0.96	1.06
rs7519574	1	34726552	G	A	-0.0959	0.07	0.1923	0.91	0.79	1.05	0.00795	0.05	0.8760	1.01	0.91	1.11
rs1343776	1	41757718	G	A	0.0262	0.03	0.3143	1.03	0.98	1.08	0.01168	0.02	0.5465	1.01	0.97	1.05
rs10789481	1	46383583	C	G	-0.0225	0.03	0.3697	0.98	0.93	1.03	0.00201	0.02	0.9132	1.00	0.97	1.04
rs2131925	1	63025942	G	T	0.0287	0.03	0.3093	1.03	0.97	1.09	-0.02345	0.02	0.2689	0.98	0.94	1.02
rs7528419	1	109817192	A	G	-0.0133	0.05	0.7825	0.99	0.90	1.08	0.04592	0.03	0.1844	1.05	0.98	1.12
rs9050	1	152079314	C	A	-0.0791	0.10	0.4229	0.92	0.76	1.12	0.04749	0.08	0.5691	1.05	0.89	1.23
rs576242124	1	152390763	G	A	0.2316	0.19	0.2329	1.26	0.86	1.84	0.45759	0.43	0.2856	1.58	0.68	3.66
rs12144049	1	152440910	C	T	0.0049	0.03	0.8721	1.00	0.95	1.07	-0.02235	0.02	0.3116	0.98	0.94	1.02
rs2807834	1	220970593	T	G	-0.0404	0.03	0.2418	0.96	0.90	1.03	-0.02376	0.03	0.3707	0.98	0.93	1.03
rs4846913	1	230294715	C	A	-0.1000	0.03	0.0012	0.90	0.85	0.96	-0.03599	0.02	0.1186	0.96	0.92	1.01
rs1260326	2	27730940	T	C	-0.0085	0.02	0.7238	0.99	0.95	1.04	0.03127	0.02	0.0891	1.03	1.00	1.07
rs35270497	2	38259872	C	T	-0.0146	0.04	0.6794	0.99	0.92	1.06	-0.00629	0.03	0.8196	0.99	0.94	1.05
rs727857	2	58981967	G	A	-0.0065	0.02	0.7860	0.99	0.95	1.04	-0.02020	0.02	0.2719	0.98	0.95	1.02
rs2710651	2	63166379	G	A	0.0242	0.02	0.3140	1.02	0.98	1.07	-0.00491	0.02	0.7900	1.00	0.96	1.03
rs6724965	2	101440151	A	G	-0.0271	0.02	0.2655	0.97	0.93	1.02	-0.02257	0.02	0.2203	0.98	0.94	1.01
rs7569755	2	118648261	G	A	0.0321	0.04	0.4454	1.03	0.95	1.12	0.03060	0.03	0.3388	1.03	0.97	1.10
rs1047891	2	211540507	C	A	0.0310	0.03	0.3673	1.03	0.96	1.10	0.01536	0.03	0.5626	1.02	0.96	1.07
rs2012736	2	234622379	C	A	-0.0162	0.03	0.6406	0.98	0.92	1.05	-0.02423	0.03	0.3901	0.98	0.92	1.03
rs9847248	3	18804655	G	A	0.0363	0.02	0.1406	1.04	0.99	1.09	0.04242	0.02	0.0215	1.04	1.01	1.08
rs1128535	3	49866392	C	T	0.0037	0.03	0.8980	1.00	0.95	1.06	-0.02960	0.02	0.1627	0.97	0.93	1.01

rs7652808	3	85603643	T	G	-0.0491	0.05	0.3146	0.95	0.87	1.05	0.00525	0.04	0.8897	1.01	0.93	1.08
rs6438900	3	125148287	C	G	-0.0645	0.03	0.0411	0.94	0.88	1.00	-0.02639	0.02	0.2721	0.97	0.93	1.02
rs34186890	3	141720712	A	G	0.0256	0.03	0.3331	1.03	0.97	1.08	-0.00984	0.02	0.6268	0.99	0.95	1.03
rs1949633	3	153758806	T	C	-0.0157	0.03	0.5394	0.98	0.94	1.03	-0.00045	0.02	0.9815	1.00	0.96	1.04
rs78649910	4	3482213	T	A	0.0173	0.03	0.5147	1.02	0.97	1.07	-0.01291	0.02	0.5683	0.99	0.94	1.03
rs4364259	4	15892159	G	A	0.0012	0.03	0.9716	1.00	0.94	1.07	0.01453	0.03	0.6059	1.01	0.96	1.07
rs4616820	4	57745481	C	T	-0.0427	0.02	0.0735	0.96	0.91	1.00	-0.01190	0.02	0.5170	0.99	0.95	1.02
rs7665631	4	70281414	A	C	-0.0044	0.03	0.8907	1.00	0.93	1.06	-0.06764	0.02	0.0062	0.93	0.89	0.98
rs3755967	4	72609398	C	T	0.0078	0.03	0.7702	1.01	0.96	1.06	-0.01371	0.02	0.4993	0.99	0.95	1.03
rs10021949	4	72764039	A	G	0.0411	0.03	0.2134	1.04	0.98	1.11	-0.03207	0.02	0.1669	0.97	0.93	1.01
rs11732896	4	88287993	G	A	0.0049	0.03	0.8500	1.00	0.95	1.06	0.00523	0.02	0.7873	1.01	0.97	1.04
rs1229984	4	100239319	T	C	0.0156	0.03	0.5829	1.02	0.96	1.07	-0.04598	0.02	0.0589	0.96	0.91	1.00
rs7718395	5	118652574	C	G	-0.0170	0.08	0.8338	0.98	0.84	1.15	-0.04212	0.06	0.4993	0.96	0.85	1.08
rs7712001	5	148020950	T	G	-0.0071	0.02	0.7731	0.99	0.95	1.04	-0.02468	0.02	0.1823	0.98	0.94	1.01
rs79666294	5	154426706	C	T	0.0905	0.08	0.2647	1.09	0.93	1.28	0.06642	0.06	0.2948	1.07	0.94	1.21
rs1321247	6	25662873	A	T	-0.0060	0.07	0.9281	0.99	0.87	1.13	0.01885	0.05	0.7197	1.02	0.92	1.13
rs742493	6	40998167	T	C	0.0968	0.05	0.0667	1.10	0.99	1.22	0.06043	0.04	0.1656	1.06	0.98	1.16
rs9476310	6	57767576	C	T	-0.0151	0.03	0.5883	0.99	0.93	1.04	-0.02576	0.02	0.1667	0.97	0.94	1.01
rs12153819	6	83773049	C	T	-0.0109	0.03	0.7540	0.99	0.92	1.06	0.01387	0.03	0.5924	1.01	0.96	1.07
rs9375037	6	121856794	A	C	0.0132	0.02	0.5958	1.01	0.96	1.06	-0.00072	0.02	0.9703	1.00	0.96	1.04
rs2248551	6	131924689	G	A	0.0193	0.03	0.4547	1.02	0.97	1.07	-0.02099	0.02	0.2789	0.98	0.94	1.02
rs10085881	7	21577960	T	C	-0.0233	0.04	0.5179	0.98	0.91	1.05	-0.00854	0.03	0.7569	0.99	0.94	1.05
rs2595644	7	43980540	G	T	-0.0457	0.02	0.0549	0.96	0.91	1.00	0.04504	0.02	0.0144	1.05	1.01	1.08
rs7784802	7	64015379	A	T	0.0447	0.03	0.1435	1.05	0.98	1.11	-0.02780	0.02	0.2441	0.97	0.93	1.02
rs41301394	7	75612803	C	T	0.0001	0.03	0.9959	1.00	0.95	1.05	0.01255	0.02	0.4986	1.01	0.98	1.05
rs1011468	7	104613791	G	A	-0.0058	0.05	0.9098	0.99	0.90	1.10	0.05522	0.04	0.2079	1.06	0.97	1.15
rs1858889	7	107117447	A	C	0.0025	0.02	0.9194	1.00	0.95	1.05	0.00106	0.02	0.9542	1.00	0.97	1.04

rs2346264	7	133536351	A	C	0.0102	0.06	0.8685	1.01	0.89	1.14	0.01491	0.05	0.7702	1.02	0.92	1.12
rs4841132	8	9183596	A	G	-0.0983	0.11	0.3544	0.91	0.74	1.12	0.13055	0.10	0.1789	1.14	0.94	1.38
rs804281	8	11611865	A	G	0.0533	0.07	0.4719	1.05	0.91	1.22	-0.07823	0.05	0.1500	0.92	0.83	1.03
rs28692966	8	25892919	G	A	0.0017	0.02	0.9441	1.00	0.95	1.05	0.00733	0.02	0.6912	1.01	0.97	1.04
rs4738684	8	59393273	A	G	-0.0295	0.03	0.3577	0.97	0.91	1.03	-0.00924	0.02	0.7010	0.99	0.95	1.04
rs12056768	8	116988527	T	G	0.0145	0.02	0.5446	1.01	0.97	1.06	-0.00952	0.02	0.5884	0.99	0.96	1.03
rs13294734	9	80710910	C	T	0.0355	0.03	0.1985	1.04	0.98	1.09	0.00872	0.02	0.6682	1.01	0.97	1.05
rs13284054	9	107669073	T	C	0.0241	0.05	0.6152	1.02	0.93	1.13	-0.00257	0.04	0.9455	1.00	0.93	1.07
rs10818769	9	125719923	C	G	-0.0457	0.03	0.1175	0.96	0.90	1.01	-0.04210	0.02	0.0583	0.96	0.92	1.00
rs532436	9	136149830	G	A	0.0372	0.03	0.1741	1.04	0.98	1.10	-0.01363	0.02	0.5020	0.99	0.95	1.03
rs2398113	10	10076429	A	G	-0.0228	0.02	0.3599	0.98	0.93	1.03	-0.02908	0.02	0.1500	0.97	0.93	1.01
rs10887718	10	82042624	C	T	-0.0056	0.04	0.8975	0.99	0.91	1.08	0.01136	0.03	0.7447	1.01	0.94	1.08
rs12775091	10	91524012	C	T	0.0048	0.02	0.8458	1.00	0.96	1.05	-0.03878	0.02	0.0440	0.96	0.93	1.00
rs2297991	10	113913222	T	C	0.0157	0.03	0.5885	1.02	0.96	1.08	0.00253	0.02	0.9053	1.00	0.96	1.05
rs2618515	11	14012957	A	G	-0.0296	0.03	0.2543	0.97	0.92	1.02	-0.00690	0.02	0.7223	0.99	0.96	1.03
rs10832254	11	14434698	A	G	0.0097	0.02	0.6845	1.01	0.96	1.06	0.00046	0.02	0.9800	1.00	0.97	1.04
rs71473824	11	71063989	C	T	0.0146	0.03	0.6177	1.01	0.96	1.07	-0.05445	0.02	0.0179	0.95	0.91	0.99
rs12803256	11	71132868	A	G	-0.0047	0.03	0.8587	1.00	0.94	1.05	0.02596	0.02	0.1810	1.03	0.99	1.07
rs964184	11	116648917	G	C	-0.0179	0.03	0.4982	0.98	0.93	1.03	-0.00776	0.02	0.7012	0.99	0.95	1.03
rs2847500	11	120114421	G	A	-0.0640	0.06	0.2738	0.94	0.84	1.05	-0.00948	0.04	0.8097	0.99	0.92	1.07
rs1871395	12	21352315	A	G	0.0363	0.03	0.1511	1.04	0.99	1.09	0.03347	0.02	0.0702	1.03	1.00	1.07
rs1396206	12	24576859	A	T	0.0102	0.07	0.8859	1.01	0.88	1.16	-0.02007	0.06	0.7154	0.98	0.88	1.09
rs11182428	12	38526387	T	C	-0.0122	0.03	0.7017	0.99	0.93	1.05	-0.03119	0.02	0.1771	0.97	0.93	1.01
rs1038165	12	68665940	C	T	0.0185	0.02	0.4445	1.02	0.97	1.07	-0.01099	0.02	0.5515	0.99	0.95	1.03
rs10859995	12	96375682	T	C	0.0150	0.02	0.5316	1.02	0.97	1.06	-0.03821	0.02	0.0384	0.96	0.93	1.00
rs73413596	12	111582630	T	C	-0.0902	0.07	0.2119	0.91	0.79	1.05	0.09787	0.05	0.0554	1.10	1.00	1.22
rs28435470	12	133067473	G	A	-0.0275	0.04	0.4690	0.97	0.90	1.05	-0.03846	0.03	0.1793	0.96	0.91	1.02

rs4580037	13	55702646	A	C	-0.0145	0.02	0.5452	0.99	0.94	1.03	0.01045	0.02	0.5695	1.01	0.97	1.05
rs2144530	14	39552484	C	T	0.0417	0.03	0.0989	1.04	0.99	1.10	0.01704	0.02	0.3781	1.02	0.98	1.06
rs2756119	14	104001517	G	A	0.0172	0.02	0.4840	1.02	0.97	1.07	0.01549	0.02	0.4226	1.02	0.98	1.05
rs1800588	15	58723675	C	T	-0.0358	0.02	0.1279	0.96	0.92	1.01	0.04075	0.02	0.0205	1.04	1.01	1.08
rs62007299	15	77711719	G	A	0.0322	0.02	0.1835	1.03	0.98	1.08	-0.01221	0.02	0.5073	0.99	0.95	1.02
rs325384	15	100229761	C	T	0.0077	0.03	0.7700	1.01	0.96	1.06	0.00477	0.02	0.8139	1.00	0.97	1.05
rs1684600	16	4594671	C	T	-0.0163	0.03	0.5499	0.98	0.93	1.04	0.01559	0.02	0.5061	1.02	0.97	1.06
rs8063706	16	11909552	A	T	0.0225	0.03	0.4272	1.02	0.97	1.08	-0.01330	0.02	0.5574	0.99	0.94	1.03
rs77924615	16	20392332	G	A	-0.0026	0.03	0.9286	1.00	0.94	1.06	-0.03110	0.02	0.1614	0.97	0.93	1.01
rs71383766	16	30930233	C	T	-0.1324	0.04	0.0011	0.88	0.81	0.95	0.00015	0.04	0.9967	1.00	0.93	1.07
rs11076175	16	57006378	A	G	0.0684	0.04	0.1198	1.07	0.98	1.17	-0.02458	0.03	0.4691	0.98	0.91	1.04
rs4575545	16	79755446	G	A	-0.0135	0.03	0.6135	0.99	0.94	1.04	-0.02323	0.02	0.2693	0.98	0.94	1.02
rs10454087	17	40735641	C	T	-0.0095	0.02	0.6858	0.99	0.95	1.04	0.00837	0.02	0.6355	1.01	0.97	1.04
rs2952289	17	66464414	C	T	0.0082	0.02	0.7379	1.01	0.96	1.06	-0.01340	0.02	0.4676	0.99	0.95	1.02
rs61698755	17	79257880	T	C	0.0013	0.03	0.9600	1.00	0.95	1.05	-0.01412	0.02	0.4666	0.99	0.95	1.02
rs8091117	18	28919794	C	A	0.0052	0.05	0.9240	1.01	0.90	1.12	0.01927	0.04	0.6402	1.02	0.94	1.11
rs4121823	18	47144223	T	A	-0.0307	0.03	0.2968	0.97	0.92	1.03	0.01661	0.02	0.4545	1.02	0.97	1.06
rs2037511	18	61366207	G	A	0.0957	0.03	0.0032	1.10	1.03	1.17	0.00829	0.02	0.7382	1.01	0.96	1.06
rs57631352	19	4338173	A	G	-0.0115	0.02	0.6293	0.99	0.94	1.04	0.02425	0.02	0.1899	1.02	0.99	1.06
rs142158911	19	11190534	G	A	0.0973	0.12	0.4211	1.10	0.87	1.40	0.25442	0.27	0.3502	1.29	0.76	2.20
rs12462826	19	11955767	G	A	-0.0952	0.07	0.1690	0.91	0.79	1.04	-0.09611	0.05	0.0555	0.91	0.82	1.00
rs58542926	19	19379549	C	T	0.0051	0.05	0.9102	1.01	0.92	1.10	-0.02968	0.04	0.4141	0.97	0.90	1.04
rs3814995	19	36342212	C	T	0.0505	0.02	0.0401	1.05	1.00	1.10	-0.00919	0.02	0.6197	0.99	0.96	1.03
rs7412	19	45412079	C	T	0.0072	0.06	0.9066	1.01	0.89	1.14	-0.06424	0.05	0.1558	0.94	0.86	1.02
rs62130059	19	48461240	C	A	0.0105	0.03	0.7137	1.01	0.96	1.07	-0.04335	0.06	0.4470	0.96	0.86	1.07
rs10426	19	51517798	G	A	-0.0072	0.04	0.8690	0.99	0.91	1.08	0.03612	0.03	0.2551	1.04	0.97	1.10
rs35313547	19	58352806	T	C	0.0330	0.04	0.4463	1.03	0.95	1.13	-0.01094	0.03	0.6976	0.99	0.94	1.05

rs2207173	20	22805061	G	A	0.0303	0.02	0.2122	1.03	0.98	1.08	0.00236	0.02	0.8981	1.00	0.97	1.04
rs6129648	20	39231118	A	G	-0.0204	0.02	0.3874	0.98	0.94	1.03	0.00252	0.02	0.8910	1.00	0.97	1.04
rs158528	20	52721956	A	G	0.0092	0.02	0.6986	1.01	0.96	1.06	0.02532	0.02	0.1675	1.03	0.99	1.06
rs2585442	20	52737123	C	G	-0.0969	0.05	0.0543	0.91	0.82	1.00	0.06294	0.04	0.0816	1.06	0.99	1.14
rs8121940	20	52742306	C	G	0.0841	0.04	0.0417	1.09	1.00	1.18	0.06667	0.03	0.0336	1.07	1.01	1.14
rs2074735	22	31535872	G	C	-0.0220	0.03	0.3890	0.98	0.93	1.03	0.00304	0.02	0.8801	1.00	0.96	1.04
rs138335	22	41227086	C	G	0.0162	0.02	0.5017	1.02	0.97	1.07	-0.01273	0.02	0.4900	0.99	0.95	1.02

Chr: Chromosome Position: Chromosomal Position (hg19) REF: Reference allele ALT: Alternative allele SE: standard error OR: odds ratio UC, LCL: Upper and lower confidence limit of 95% confidence interval

Supplemental Table 3. Mendelian Randomization estimates between plasma vitamin D concentrations and total or colorectal cancer using a limited number of SNPs

	Total cancer			Colorectal Cancer		
	IVW	MR-Egger	Weighted median	IVW	MR-Egger	Weighted median
2 SNPs (p<5*10^-8) and 1 SNP (p<5*10^-6)[1]	0.91 (0.63-1.31)	0.93 (0.22-3.99)	0.91 (0.61-1.36)	1.14 (0.87-1.51)	0.73 (0.25-2.17)	1.10 (0.80-1.51)
5 SNPs (F-statistics>10)[2]	0.78 (0.53-1.16)	1.78 (0.75-4.21)	0.91 (0.62-1.33)	1.11 (0.86-1.44)	1.12 (0.58-2.16)	1.10 (0.80-1.50)

1 Three SNPs (p<5*10^-8) were rs3755967, rs10832254 and rs12803256 (p<5*10^-6). We included three instead of two SNPs to perform MR. Details of these SNPs were in Table 2.

2 Five SNPs (F-statistics>10) were rs3755967, rs10832254, rs12803256, rs2144530 and rs3814995.

Supplemental Table 4. Mendelian Randomization estimates between plasma vitamin D concentrations and total or colorectal cancer excluding overlapped cohort[1]

MR Method	Odds Ratio (95% CI)	p value
Total cancer		
IVW	0.98 (0.60-1.59)	0.92
MR Egger	0.95 (0.49-1.83)	0.88
Egger intercept	-	0.91
Weighted median	1.00 (0.50-2.00)	0.99
Colorectal cancer		
IVW	0.98 (0.78-1.24)	0.89
MR Egger	0.99 (0.72-1.36)	0.95
Egger intercept	-	0.95
Weighted median	1.06 (0.78-1.45)	0.70

1. We excluded JPHC-base from SNP-total cancer and SNP-colorectal cancer association, and calculated estimates.

Supplemental Table 5. Detailed information of included studies

Study	Description
JPHC-base	The Japan Public Health Centre–based Prospective (JPHC) Study was started in 1990 (cohort I) and 1993-1994 (cohort II). Participants were Japanese and residents in 11public health centers in Japan. At the baseline survey, the age of participants was 40-59 in 1990 (cohort I) and 40-69 years in 1993 (cohort II). The JPHC Study has been described in detail previously [Jpn J Clin Oncol.2014;44:777-821]. A case-cohort design ("JPHC-base" in this study) was applied to examine the association between the study participants' SNPs and total and colorectal cancer risk. In this case-cohort study, 33,736 subjects from nine public health centers across Japan, who responded to the baseline questionnaire and provided blood samples during the health check-up, were included. Measurement of Plasma Vitamin D was described elsewhere [BML.2018;360]. Participants who measured Vitamin D were randomly selected from cohort samples. The study was approved by the institutional review board of the National Cancer Centre, Japan, and provided eligible subjects with the option of refusing participation in the research.
JPHC-5year	The JPHC Study has been described above. A case-cohort design ("JPHC-5year" in this study) study was used to investigate the association between the study participants' SNPs and total and colorectal cancer risk. 10,950 participants who answered the five year-follow up questionnaire and provided blood were included. These participants were not included in "JPHC-base" described above. The study was approved by the institutional review board of the National Cancer Centre, Japan, and provided eligible subjects with the option of refusing participation in the research.
J-MICC	In the J-MICC Study [J Epidemiol.2021;31:660-668], participants were followed for cancer incidence from the baseline survey through 2015. Cancer cases were ascertained by using population- and/or hospital-based cancer registries, surveys in principal hospitals in study areas, and self-reports by participants followed by confirmation with medical records. Colorectal cancer was defined using the ICD-O-3 code (C18, C19, and C20). Genotyping with Illumina Asian Screening Array was conducted in cancer cases and selected non-cancer participants. For the SNP exposure association, subjects for the analysis were selected from the participants in the second survey in one area of the J-MICC Study (Daiko Study), and serum vitamin D was measured. The J-MICC Study, including genotyping, was approved by the ethics committee of the Nagoya University Graduate School of Medicine (Approval No.: 2010-0939), Nagoya, Japan, and written informed consent was obtained from all the participants in the present study.
NAGANO	A hospital-based case-control study of gastrointestinal cancer ("NAGANO Study") was conducted between October 1998 and March 2002, in four hospitals, in Nagano Prefecture, Japan [Nutr Cancer. 2003;46:138-47]. The eligible cases included as colorectal cancer patients were diagnosed during the survey period in the hospitals. Participants were aged 20–74 years. The study included 121 colorectal cancer patients. Controls were selected from the medical check-up examinees in the four respective hospitals and matched for sex, age (within 3 years), and residential area during the study period. Eligible healthy controls were confirmed that they did not have any cancer during the medical check-up including upper gastrointestinal endoscopy or X-ray, fecal occult blood test, and abdominal ultrasound. The study obtained a written informed consent from all cases and controls and was approved by the Institutional Review Board of the National Cancer Centre, Tokyo, Japan.
HERPACC	Hospital-based Epidemiologic Research Program at Aichi Cancer Center (HERPACC)-2 study was conducted between January 2001 and November 2005 at Aichi Cancer Center. Detail of HERPACC is described elsewhere [Asian Pac J Cancer Prev 2001;2: 99-107]. Briefly, all first-visit outpatients (n=29,736) during the study period were asked to complete self-administered questionnaire and provide blood samples. Of these, 28,776 (96.7%) participated the study with written informed consent and 13,824 subjects (48.0% of participants) further provided blood samples. All the subjects were asked to fill out self-administered questionnaire. Among13,824, 7,053 subjects were confirmed to have<br>no detectable cancer and no history of neoplasia within one-year window period from participation and were used for pool for candidates for non-cancer controls. The rest were considered as cancer cases, and further categorized into incident cases and prevalent cases. A total of 163 incident colorectal cancer cases and 3,819 non cancer controls who had been randomly selected and genotyped by Illumina Human Core Exome 12/24 were used for this case-control study. The study protocol was approved by ethics committees at Aichi Cancer Center, Nagoya, Japan.
BBJ	There is a public summary-data of GWAS in the Biobank Japan (BBJ) study in the Japanese ENcyclopedia of GEnetic associations by Riken (JENGER) (http://jenger.riken.jp/result). This study's description for the association between Japanese SNPs and colorectal cancer was shown previously

	[Carcinogenesis. 2018;39:652-60].

JPHC=Japan Public Health Centre–based Prospective, J-MICC=Japan Multi-Institutional Collaborative Cohort, J-MICC=Japan Multi-Institutional Collaborative Cohort, HERPACC=Hospital-based Epidemiologic Research Program at Aichi Cancer Center, BBJ=BioBank Japan.

Supplemental Table 6. Genotyping, imputation and statistical analysis method in participated studies

	Study name	Genotyping					Imputation		Statistical analysis	
		Platform	QC				Software	Reference	Software	Covariates
			MAF	Callrate	HWE	Other criteria				
Exposure	JPHC	Illumina HumanOmni2.5 /Express/ExpressExome	≥1%	≥98%	≥1 x 10^{-6}	Sex mismatches, related samples (IBD≥0.1875)	SHAPEIT2 IMPUTE2	1000G phase3v5; individuals of ALL	SAS	age, sex, seasons, area, top 5PC
	J-MICC	Illumina HumanOmni ExpressExome	≥1%	≥98%	≥1 x 10^{-6}	Sex mismatches, related samples (IBD≥0.1875)	SHAPEIT2 IMPUTE2	1000G phase3v5; individuals of ALL	SNPTEST	age, sex, seasons top5PC
Outcome (Colorectal Cancer)	JPHC-base	Illumina HumanOmni2.5 /Express/ExpressExome	≥1%	≥98%	≥1 x 10^{-6}	Sex mismatches, related samples (IBD≥0.1875)	SHAPEIT2 IMPUTE2	1000G phase3v5; individuals of ALL	SNPTEST	age, sex, top5PC
	JPHC-5 year	Illumina Human OmniExpressExome	≥1%	≥98%	≥1 x 10^{-6}		SHAPEIT2 IMPUTE2	1000G phase3v5; individuals of ALL	SNPTEST	age, sex, top 10PC
	J-MICC	Illumina Asian Screening Array	≥1%	≥98%	≥1 x 10^{-6}	Sex mismatches, related samples (IBD≥0.1875)	SHAPEIT2 minimac3	1000G phase3v5; individuals of ALL	SNPTEST	age, sex, top 5PC
	NAGANO	Illumina1M-duo	≥1%	≥98%	≥1 x 10^{-6}	Sex mismatches, related samples (IBD≥0.1875)	SHAPEIT2 IMPUTE2	1000G phase3v5; individuals of ALL	SNP TEST	age, sex, top 3PC
	HERPACC	Illumina Human CoreExome 12/24	≥1%	≥98%	≥1 x 10^{-6}	Sex mismatches, related samples (IBD≥0.1875)	SHAPEIT2 minimac3	1000G phase3v5; individuals of ALL	PLINK2	age, sex, top 5PC
	J-MICC	Illumina Asian Screening Array	≥1%	≥98%	≥1 x 10^{-6}	Sex mismatches, related samples (IBD≥0.1875)	SHAPEIT2 minimac3	1000G phase3v5; individuals of ALL	SNP TEST	age, sex, top 5PC
	BBJ	Illumina OmniExpressExome /OmniExpress/Human Exome	≥1%	≥99%	≥1 x 10^{-6}	ND	MaCH minimac	1000G phase1; individuals of JPT/CHS/CHD	ND	age, sex, top 2PC

QC=quality control, IBD=identity-by-descent, MAF= minor allele frequency, HWE=Hardy-Weinberg equilibrium, JPHC=Japan Public Health Centre–based Prospective, J-MICC=Japan Multi-Institutional Collaborative Cohort, J-MICC=Japan Multi-Institutional Collaborative Cohort, HERPACC=Hospital-based Epidemiologic Research Program at Aichi Cancer Center, BBJ=BioBank Japan. For total cancer, information from JPHC-base and JPHC-5 year was used and calculated with weighted Cox analysis using SAS.

Supplemental Table 7. STROBE-MR checklist for the report using Mendelian randomization method

Item No.	Section		Checklist item	Page No.	Relevant text from manuscript
1	**TITLE and ABSTRACT**		Indicate Mendelian randomization (MR) as the study's design in the title and/or the abstract if that is a main purpose of the study	1	Association of 25-hydroxyvitamin D with risk of overall and colorectal cancer among Japanese: A Mendelian Randomization approach
	**INTRODUCTION**				
2	**Background**		Explain the scientific background and rationale for the reported study. What is the exposure? Is a potential causal relationship between exposure and outcome plausible? Justify why MR is a helpful method to address the study question	4-5	Introductionn paragraph2-4
3	**Objectives**		State specific objectives clearly, including pre-specified causal hypotheses (if any). State that MR is a method that, under specific assumptions, intends to estimate causal effects	4-5	Introductionn paragraph 4.5
	**METHODS**				
4	**Study design and data sources**		Present key elements of the study design early in the article. Consider including a table listing sources of data for all phases of the study. For each data source contributing to the analysis, describe the following:	10-11	Supplemental Table 6,7
		a)	Setting: Describe the study design and the underlying population, if possible. Describe the setting, locations, and relevant dates, including periods of recruitment, exposure, follow-up, and data collection, when available.		Supplemental Table 6
		b)	Participants: Give the eligibility criteria, and the sources and methods of selection of participants. Report the sample size, and whether any power or sample size calculations were carried out prior to the main analysis	10	Supplemental Table 6, Table1, Method "The data source of MR analysis in Japanese"
		c)	Describe measurement, quality control and selection of genetic variants	9	Supplemental Table 6, Supplemental Table 7, Method "Methods for selecting SNPs associated with Vitamin D"
		d)	For each exposure, outcome, and other relevant variables, describe methods of assessment and diagnostic criteria for diseases		Supplemental Table 6 (Details were in the reference paper in each study)
		e)	Provide details of ethics committee approval and participant informed consent, if relevant		Supplemental Table 6
5	**Assumptions**		Explicitly state the three core IV assumptions for the main analysis (relevance, independence and exclusion restriction) as well assumptions for any additional or	9	Method paragraph 1

		sensitivity analysis		
6	**Statistical methods: main analysis**	Describe statistical methods and statistics used	11	Method "Statistical Analysis"
	a)	Describe how quantitative variables were handled in the analyses (i.e., scale, units, model)	10	Method "The data source of MR analysis in Japanese"
	b)	Describe how genetic variants were handled in the analyses and, if applicable, how their weights were selected	11	Method "Statistical Analysis"
	c)	Describe the MR estimator (e.g. two-stage least squares, Wald ratio) and related statistics. Detail the included covariates and, in case of two-sample MR, whether the same covariate set was used for adjustment in the two samples	11	Method "Statistical Analysis"
	d)	Explain how missing data were addressed	-	
	e)	If applicable, indicate how multiple testing was addressed	-	
7	**Assessment of assumptions**	Describe any methods or prior knowledge used to assess the assumptions or justify their validity	11	Method "Statistical Analysis"
8	**Sensitivity analyses and additional analyses**	Describe any sensitivity analyses or additional analyses performed (e.g. comparison of effect estimates from different approaches, independent replication, bias analytic techniques, validation of instruments, simulations)	11	Method "Statistical Analysis"
9	**Software and pre-registration**			
	a)	Name statistical software and package(s), including version and settings used	11	Method "Statistical Analysis"
	b)	State whether the study protocol and details were pre-registered (as well as when and where)	10	Method "The data source of MR analysis in Japanese"
	**RESULTS**			
10	**Descriptive data**			
	a)	Report the numbers of individuals at each stage of included studies and reasons for exclusion. Consider use of a flow diagram	5	Result paragraph1
	b)	Report summary statistics for phenotypic exposure(s), outcome(s), and other relevant variables (e.g. means, SDs, proportions)		Table1, Supplemental table1,2
	c)	If the data sources include meta-analyses of previous studies, provide the assessments of heterogeneity across these studies	-	
	d)	For two-sample MR:		Table1, Supplemental table1,2

			i. Provide justification of the similarity of the genetic variant-exposure associations between the exposure and outcome samples<br>ii. Provide information on the number of individuals who overlap between the exposure and outcome studies		
11	**Main results**				
		a)	Report the associations between genetic variant and exposure, and between genetic variant and outcome, preferably on an interpretable scale		Supplemental table1,2
		b)	Report MR estimates of the relationship between exposure and outcome, and the measures of uncertainty from the MR analysis, on an interpretable scale, such as odds ratio or relative risk per SD difference		Result paragraph 4, Table 3
		c)	If relevant, consider translating estimates of relative risk into absolute risk for a meaningful time period		
		d)	Consider plots to visualize results (e.g. forest plot, scatterplot of associations between genetic variants and outcome versus between genetic variants and exposure)		Figure1
12	**Assessment of assumptions**				
		a)	Report the assessment of the validity of the assumptions	6	Result paragraph 4
		b)	Report any additional statistics (e.g., assessments of heterogeneity across genetic variants, such as I^2, Q statistic or E-value)	6	Result paragraph 4, Supplemental table 1
13	**Sensitivity analyses and additional analyses**				
		a)	Report any sensitivity analyses to assess the robustness of the main results to violations of the assumptions	6	Result paragraph 4, Supplemental table 3,4, Table2
		b)	Report results from other sensitivity analyses or additional analyses	6	Result paragraph 4, Supplemental table 3,4, Table2
		c)	Report any assessment of direction of causal relationship (e.g., bidirectional MR)	-	Because we are based on the prospective studies and baseline Vitamin D was exposure in our hypothesis, we did not conduct bidirectional MR in this analysis.
		d)	When relevant, report and compare with estimates from non-MR analyses	-	
		e)	Consider additional plots to visualize results (e.g., leave-one-out analyses)	-	

DISCUSSION

14	**Key results**	Summarize key results with reference to study objectives	7	Discussion paragraph 1
15	**Limitations**	Discuss limitations of the study, taking into account the validity of the IV assumptions, other sources of potential bias, and imprecision. Discuss both direction and magnitude of any potential bias and any efforts to address them	9	Limitation part
16	**Interpretation**			
	a)	Meaning: Give a cautious overall interpretation of results in the context of their limitations and in comparison with other studies	7，8	Discussion paragraph2、3
	b)	Mechanism: Discuss underlying biological mechanisms that could drive a potential causal relationship between the investigated exposure and the outcome, and whether the gene-environment equivalence assumption is reasonable. Use causal language carefully, clarifying that IV estimates may provide causal effects only under certain assumptions	8	Discussion paragraph4
	c)	Clinical relevance: Discuss whether the results have clinical or public policy relevance, and to what extent they inform effect sizes of possible interventions	-	
17	**Generalizability**	Discuss the generalizability of the study results (a) to other populations, (b) across other exposure periods/timings, and (c) across other levels of exposure	9	Discussion limitation part
	**OTHER INFORMATION**			
18	**Funding**	Describe sources of funding and the role of funders in the present study and, if applicable, sources of funding for the databases and original study or studies on which the present study is based	12	
19	**Data and data sharing**	Provide the data used to perform all analyses or report where and how the data can be accessed, and reference these sources in the article. Provide the statistical code needed to reproduce the results in the article, or report whether the code is publicly accessible and if so, where	12	
20	**Conflicts of Interest**	All authors should declare all potential conflicts of interest	12	
